# Skeletal muscle Nur77 and NOR1 insulin responsiveness is blunted in obesity and type 2 diabetes but improved after exercise training

**DOI:** 10.14814/phy2.14042

**Published:** 2019-03-25

**Authors:** Jacob T. Mey, Thomas P. J. Solomon, John P. Kirwan, Jacob M. Haus

**Affiliations:** ^1^ Department of Kinesiology and Nutrition University of Illinois Chicago Chicago Illinois; ^2^ School of Sport, Exercise, and Rehabilitation Sciences University of Birmingham Birmingham United Kingdom; ^3^ Metabolic Translational Research Center Endocrinology & Metabolism Institute Cleveland Clinic Cleveland Ohio; ^4^ Integrative Physiology and Molecular Medicine Laboratory Pennington Biomedical Research Center Baton Rouge Louisiana; ^5^ School of Kinesiology University of Michigan Ann Arbor Michigan

**Keywords:** Insulin resistance, nutrient metabolism, obesity, type 2 diabetes

## Abstract

Obesity and type 2 diabetes (T2DM) are characterized by a blunted metabolic response to insulin, and strongly manifests in skeletal muscle insulin resistance. The orphan nuclear receptors, Nur77 and NOR1, regulate insulin‐stimulated nutrient metabolism where *Nur77* and *NOR1* gene expression is increased with acute aerobic exercise and acute insulin stimulation. Whether Nur77 or NOR1 are associated with the insulin‐sensitizing effects of chronic aerobic exercise training has yet to be elucidated. Fourteen lean healthy controls (LHC), 12 obese (OB), and 10 T2DM individuals (T2DM) underwent hyperinsulinemic‐euglycemic clamps with skeletal muscle biopsies. Muscle was analyzed for Nur77 and NOR1 gene and protein expression at basal and insulin‐stimulated conditions. Furthermore, a subcohort of 18 participants (OB,* n* = 12; T2DM,* n* = 6) underwent a 12‐week aerobic exercise intervention (85% HR_max_, 60 min/day, 5 days/week). In response to insulin infusion, LHC increased protein expression of Nur77 (8.7 ± 3.2‐fold) and NOR1 (3.6 ± 1.1‐fold), whereas OB and T2DM remained unaffected. Clamp‐derived glucose disposal rates correlated with Nur77 (*r* ^2^ = 0.14) and NOR1 (*r* ^2^ = 0.12) protein expression responses to insulin, whereas age (Nur77: *r* ^2^ = 0.22; NOR1: *r* ^2^ = 0.25) and BMI (Nur77: *r* ^2^ = 0.22; NOR1: *r* ^2^ = 0.42) showed inverse correlations, corroborating preclinical data. In the intervention cohort, exercise improved Nur77 protein expression in response to insulin (PRE: −1.2 ± 0.3%, POST: 6.2 ± 1.5%). Also, insulin treatment of primary human skeletal muscle cells increased Nur77 and NOR1 protein. These findings highlight the multifactorial nature of insulin resistance in human obesity and T2DM. Understanding the regulation of Nur77 and NOR1 in skeletal muscle and other insulin‐sensitive tissues will create opportunities to advance therapies for T2DM.

## Introduction

Gene microarray studies in the past decade have brought about numerous putative therapeutic targets, especially related to obesity and type 2 diabetes (T2DM). In skeletal muscle, orphan nuclear receptors represent a group of targets that have been consistently identified in these screens. In human skeletal muscle, two members of the orphan nuclear receptor family, Nur77 and NOR1 (also known as NR4A1 and NR4A3, respectively), received particular attention due to acute gene inductions in response to insulin (Wu et al. 2007) and aerobic exercise (Mahoney and Tarnopolsky 2005; Mahoney et al. 2005). Nur77 and NOR1 control important pathways dysregulated in insulin resistance, like glucose (Kanzleiter et al. 2009, 2010) and lipid metabolism (Maxwell et al. 2005; Chao et al. 2009, 2012), and are therefore promising targets for developing therapeutics for chronic diseases, including obesity and T2DM. Encouraging evidence from preclinical mouse models show Nur77 and NOR1 overexpression increases glucose uptake (Kanzleiter et al. 2009) and fat oxidation (Chao et al. 2012). In agreement, mouse knockout models display impaired insulin signaling, ectopic fat accumulation, and increased susceptibility to diet‐induced obesity (Chao et al. 2009). Furthermore, Nur77 is reduced in the skeletal muscle of murine models of insulin resistance (Pei et al. 2006; Fu et al. 2007; Lessard et al. 2009) along with obese humans (Kanzleiter et al. 2010). Additionally, a defining characteristic in insulin resistance and T2DM is the dysregulation of skeletal muscle genes and proteins in response to insulin stimulation. Both Nur77 and NOR1 gene and protein expression are acutely induced by insulin (Rome et al. 2003; Fu et al. 2007; Wu et al. 2007; Coletta et al. 2008; Kanzleiter et al. 2010) in healthy skeletal muscle. However, the acute response of Nur77 and NOR1 gene and protein expression in response to insulin has not been characterized in the context of obesity, insulin resistance or T2DM.

Intensive lifestyle intervention, such as aerobic exercise training, is a well‐documented countermeasure to obesity, insulin resistance, and T2DM. Chronic aerobic exercise imparts robust benefits on insulin sensitivity, along with glucose and lipid metabolism (O'Leary et al. 2006; Solomon et al. 2008; Haus et al. 2009, 2010, 2011; Wang et al. 2009). These exercise‐mediated metabolic improvements are driven by chronic adaptations in skeletal muscle, for example, skeletal muscle gene and protein responses to insulin stimulation (Borghouts and Keizer 2000; Perry et al. 2010). It was recently suggested that these skeletal muscle responses may be regulated by NOR1 (Pearen and Muscat 2018). Indeed, aerobic exercise acutely increases both Nur77 and NOR1 (Mahoney et al. 2005) in healthy individuals. Furthermore, evidence from skeletal muscle cell culture (Maxwell et al. 2005), preclinical models (Pearen et al. 2008; Kawasaki et al. 2009; Lessard et al. 2011) and humans (Mahoney et al. 2005; Lewis et al. 2010) demonstrate that aerobic exercise (or an exercise effector in cell culture, ß‐adrenergic stimulation) increases Nur77 and NOR1 gene and protein expression. Despite this evidence, well‐designed metabolic studies investigating Nur77 and NOR1 gene and protein expression in skeletal muscle have failed to demonstrate effect in the context of obesity, insulin resistance and T2DM, or following chronic adaptations to therapeutic lifestyle interventions.

The purpose of this study was twofold. First, we sought to characterize Nur77 and NOR1 protein expression in response to insulin stimulation in human skeletal muscle. We hypothesized that individuals with obesity or T2DM would have reduced skeletal muscle Nur77 and NOR1 gene and protein expression in response to insulin stimulation, related to the subject's degree of insulin resistance. Second, we examined the effect of an intensive lifestyle intervention, involving aerobic exercise training and dietary control, on skeletal muscle Nur77 and NOR1 gene and protein expression. We hypothesized that aerobic exercise training would normalize basal Nur77 and NOR1 gene and protein expression while improving the Nur77 and NOR1 protein response to insulin stimulation. We expected these changes would be related to improvements in insulin sensitivity. Finally, we utilized primary human skeletal muscle cell cultures to examine the effects of isolated insulin treatment on Nur77 and NOR1 protein expression as a mechanistic proof‐of‐principle validation experiment.

## Methods and Materials

### Study overview

This study employed both cross‐sectional and longitudinal study designs. The cross‐sectional investigation included the study of 12 obese (OB) and 10 age‐ and BMI‐matched newly diagnosed insulin naïve T2DMs, along with 14 younger lean healthy controls (LHC). The LHC group serves as a model of ideal skeletal muscle health and provides a reference group for comparison with the OB and T2DM participants. Comprehensive metabolic characterization included an oral glucose tolerance test (OGTT), hyperinsulinemic‐euglycemic clamp and skeletal muscle biopsy, at basal and insulin‐stimulated conditions of the clamp. A subsection of participants from the cross‐sectional cohort (12 OB and 6 T2DM) underwent an intensive lifestyle intervention involving aerobic exercise training. Metabolic characterization was repeated after completion of the intervention. Skeletal muscle tissue samples were probed for Nur77 and NOR1 gene and protein expression, and skeletal muscle metabolic regulatory proteins. Subject characteristics and metabolic data from a cohort of these participants was previously published, however, this is the first reporting of Nur77 and NOR1 in these participants (Solomon et al. 2010; Williamson et al. 2015; Mahmoud et al. 2016). Finally, primary human skeletal muscle myoblasts were differentiated into myotubes and used for mechanistic validation of target proteins.

### Subjects

Participants were recruited from the Cleveland, OH, and Chicago, IL, metropolitan areas. All subjects were screened via health history, medical exam, resting EKG, and fasting blood chemistry in the Clinical Research Centers of MetroHealth Medical Center, the Cleveland Clinic, and the University of Illinois at Chicago. Individuals were excluded if they used nicotine, experienced > 2 kg weight change within the previous 6 months, or had evidence of hematological, renal, hepatic, or cardiovascular disease. Body composition was measured by dual‐energy X‐ray absorptiometry (model iDXA; Lunar, Madison, WI). Glucose tolerance was characterized by a 75 g OGTT following a standard 10‐ to 12‐hour fasting period; LHCs were excluded with a 2‐h OGTT glucose > 140 mg/dL, whereas OB were excluded with a 2‐h OGTT glucose > 200 mg/dL according to American Diabetes Association clinical practice standards. All studies were approved by the Institutional Review Boards of MetroHealth Medical Center, the Cleveland Clinic and the University of Illinois at Chicago and performed in accordance with the Declaration of Helsinki. Written informed consent was obtained from all research participants prior to initiating study procedures.

### Metabolic control procedures

For 3 days prior to metabolic testing, subjects were counseled to eat a balanced diet that contained at least 200 g of carbohydrate daily to stabilize muscle and liver glycogen stores (Solomon et al. 2009). Subjects also provided a 3‐day dietary recall, recording the time, volume, and content of food consumed. Subjects were asked to refrain from consuming foods and beverages that contained caffeine 24 h prior to testing, and from consuming alcoholic beverages 48 h prior to testing. Additionally, subjects were instructed to refrain from physical activity outside of their normal activities of daily living for 48 hours prior to metabolic testing. The evening preceding metabolic tests, subjects were provided a balanced meal containing 55% carbohydrate, 35% fat, and 10% protein. After the meal, subjects fasted overnight for 10–12 h. All participants were asked to withhold medications known to affect exercise performance and insulin sensitivity the evening prior to metabolic testing. This approach has been used successfully to control for the influence of diet and physical activity in the study of insulin sensitivity and metabolism (Haus et al. 2010, 2011; Kelly et al. 2012).

### Assessment of insulin sensitivity and substrate utilization

Whole‐body insulin sensitivity was assessed using the hyperinsulinemic (40 mU/m^2^/min)‐euglycemic (5 mmol/L) clamp procedure, performed as previously detailed (Solomon et al. 2010; Williamson et al. 2015). Blood glucose was measured every 5 min via YSI glucose‐lactate analyzer (YSI 2300; STAT Plus, Yellow Springs, OH) and was clamped at 90 mg/dL by variable glucose infusion (20% dextrose). Glucose disposal rates (GDR; mg/kg/min) were calculated as the mean rate obtained during steady‐state insulin‐stimulated conditions and whole‐body insulin sensitivity is reported as “M/I” (mg/kg/min/*μ*U.mL) and calculated from GDR normalized to the steady‐state insulin concentration during 90–120 min of the clamp procedure. Indirect calorimetry was performed prior to starting the clamp (basal, postabsorptive metabolism) and during 90–120 min (insulin‐stimulated metabolism) of the clamp procedure. Expired air was continuously sampled for a minimum of 20 min with an automated system (Viasys HealthCare, Yorba Linda, CA; Parvo Medics, Sandy, UT) in a semidarkened, thermoneutral (22 ± 1°C) environment under a ventilated hood with subjects laying supine. The equations of Weir (1949) and Frayn (1983) were used to calculate energy expenditure (BMR) and substrate oxidation rates (carbohydrate oxidation, COX; fat oxidation, FOX). In addition, overnight, timed urinary nitrogen excretion measurements (Roche Modular Diagnostics, Indianapolis, IN) were also made to correct for protein metabolism (Frayn 1983). Nonoxidative glucose metabolism was calculated as GDR minus insulin‐stimulated carbohydrate oxidation. Pre and postintervention metabolic flexibility was calculated as the response of postabsorptive substrate metabolism to hyperinsulinemic conditions, for example, insulin‐stimulated respiratory exchange ratio (RER) minus postabsorptive RER (Solomon et al. 2009).

### Skeletal muscle biopsy

Skeletal muscle biopsies (~200 mg) of the *vastus lateralis* were obtained under local anesthesia (Lidocaine HCl 1%), (Trappe et al. 2013) using a 5 mm Bergstrom needle with suction (Bergstrom 1975; Evans et al. 1982) during the baseline (0 min) and insulin‐stimulated (120 min) periods of the hyperinsulinemic‐euglycemic clamp procedure (Williamson et al. 2015; Mahmoud et al. 2016). Muscle tissue was blotted, trimmed of adipose and connective tissue, immediately flash frozen in liquid nitrogen, and subsequently stored at −80°C for later analysis. An aliquot of muscle tissue (~20 mg) was placed into RNALater (AMBION, Inc., Austin, TX) and stored at ‐20°C until processing for extraction of total RNA.

### Aerobic exercise training intervention

A subcohort of 18 participants (OB, *n* = 12; T2DM, *n* = 6) were enrolled in a 12‐week intensive lifestyle intervention. Insulin sensitivity, aerobic fitness, glucose and lipid metabolism, and skeletal muscle gene, and protein targets were measured before and after the intervention. Stringent control procedures included a 2‐day screening procedure, 3‐day inpatient stays for pre and posttesting procedures, 12 weeks of fully supervised exercise, and a full diet provision (isocaloric) during the intervention period; all of which have been detailed previously (Solomon et al. 2010). The exercise intervention provided 60 minutes of aerobic exercise 5 days/wk for 12 weeks (treadmill walking and cycle ergometry) at 70% *V*O_2_max (85% of the HR_max_). A 5‐min warm up and cool down were performed during each exercise bout and are included within the 60‐min total. Subjects also performed stretching exercises for the quadriceps, hamstrings, and gastrocnemius before and after each session. A maximal exercise test was used to assess cardiovascular fitness as well as to adjust exercise intensities throughout the 12‐week intervention to maintain training intensity at 70% *V*O_2_max. To avoid acute effects of exercise on insulin sensitivity, exercise testing was performed ~48 h before metabolic testing and sample collections.

### Maximal oxygen consumption

Maximal oxygen consumption (*V*O_2max_) and maximum heart rate (HR_max_) were determined from an incremental treadmill test using a modified Bruce protocol. Expired air was continuously sampled online with the use of an automated system (Jaeger Oxycon Pro; Viasys, Yorba Linda, CA). Each test was conducted by an exercise physiologist. The test was considered successful if ≥ 3 of the following criteria were reached: plateau in oxygen consumption with increasing workload, volitional fatigue, heart rate ≤ 10 beats/min of the age‐predicted maximum, or a respiratory exchange ratio > 1.10 (Solomon et al. 2010).

### Analytical assessments

For immunoblotting, approximately 10–15 mg (wet weight) of frozen muscle tissue was homogenized by ceramic beads (lysing matrix D; FastPrep^®^‐24 homogenizer, MP Biomedicals, Santa Ana, CA) in 20 volumes of ice‐cold lysis buffer (Cell Signaling Technology, Beverly, MA) supplemented with a protease and phosphatase inhibitor cocktail (MS‐SAFE, Sigma Aldrich, St Louis MO). Total protein concentration was determined via BCA Protein Assay (Pierce Biotechnology, Rockford, IL) and proteins were separated via 10% SDS‐PAGE and transferred to either PVDF or nitrocellulose membranes (Mey et al. 2017; Mulya et al. 2017). Blotting conditions were optimized individually for each protein of interest using the antibodies as follows: Nur77 (sc‐7014, Santa Cruz Biotechnologies, Dallas, TX; ab109180, Abcam, Cambridge, MA), NOR1 (sc‐393903, Santa Cruz Biotechnologies; ab56340, Abcam,), ß‐Actin (sc‐130656, Santa Cruz Biotechnologies), and GAPDH (2118S; Cell Signaling Technology, Beverly, MA). Secondary antibodies were selected for the appropriate species and imaging procedures. Chemiluminescence (GE Healthcare, Piscataway, New Jersey) or near‐infrared fluorescence imaging (Odyssey Clx, LI‐COR Biosciences, Lincoln, NE) was used for protein visualization. GAPDH and ß‐Actin were used as loading controls.

For gene expression, skeletal muscle total RNA was extracted using the RNeasy Fibrous Tissue Mini Kit (Qiagen, Germantown, MD) with modifications for extraction from skeletal muscle stored in RNALater. RNA concentration and quality were determined by NanoDrop spectrophotometer (Thermo Scientific, Wilmington, Delaware). Total RNA (1 *μ*g) was reverse transcribed to cDNA (Clontech, Mountain View, CA) and target genes were analyzed using quantitative real‐time PCR via Brilliant SYBR Green DNA intercalation (Agilent, Santa Clara, CA). Human GAPDH primer pairs were purchased from BIOMOL International (Plymouth Meeting, PA) and all other primer pairs were designed and purchased through Integrated DNA Technologies (Coralville, IA). For human NOR1, previous research defined four gene variants that encode three distinct protein isoforms (A, B, C) (Ohkura et al. 1998). The individual physiological functions of these isoforms remain unknown in human skeletal muscle, and thus all isoforms were examined via individualized primers for each isoform. Primer sequences and accession number details are outlined in Table 1. GAPDH was used for normalization as a housekeeping gene and gene expression was determined using the 2^−ΔCT^ relative quantification method.

**Table 1:** Primers

Gene	Forward Primer (5′ – 3′)	Reverse Primer (5′‐3′)	Accession #
Nur77	ACT GGA TTG ACA GTA TCC TGG C	CCA ACA GAC GTG ACA GGC A	NM_002135.3
NOR1‐A	ATG AGG TGG GAG AAA GCA ACC ACA	TGG CCA AGG GAG AGA GTT GAC ATT	NM_173198
NOR1‐B	TTG GAA ATG TGG ATA TGC CCT GCG	AAG GTC CAT GGT CAG CTT GGT GTA	NM_173200
NOR1‐C	GAT TCC TGG TGG TTG TGC CAA TGA	TGA GGC TCA CCC AGA TGG TTT GAA	NM_173199
PGC1*α*	CAA GCC AAA CCA ACA ACT TTA TCT CT	CAC ACT TAA GGT GCG TTC AAT AGT C	NM_013261
PDK4	TGA GAC TCG CCA ACA TTC TG	CCA AAT CCA TCA GGC TCT GT	NM_002612.3
Lipin1*α*	TAA TGC CAG TTA CGA CGC TG	CAT TCC TCG ATT GAC CCT GT	NM_145693.1
GAPDH	TGA TGA CAT CAA GAA GGT GGT GAA G	TCC TTG GAG GCC ATG TGG GCC AT	NM_002046

Nur77, orphan nuclear receptor NR4A1; NOR1‐A, orphan nuclear receptor NR4A3 isoform A; NOR1‐B, orphan nuclear receptor NR4A3 isoform B; NOR1‐C, orphan nuclear receptor NR4A3 isoform C; PGC1*α*, peroxisome proliferator‐activated receptor (PPAR) *γ* coactivator‐1*α*; PDK4, pyruvate dehydrogenase kinase‐4; GAPDH, glyceraldehyde‐3‐phosphate dehydrogenase.

### Cell culture validation study

Cell culture studies have previously shown that a putative mechanism by which acute exercise induces Nur77 and NOR1 gene and protein expression is through ß‐adrenergic stimulation, which can be recapitulated through isoprenaline treatment (Pearen et al. 2006, 2008; Myers et al. 2009). Whole‐body insulin stimulation has also been shown to induce a ß‐adrenergic response (Creager et al. 1985), thus leaving the independent effects of insulin on Nur77 and NOR1 unknown in human skeletal muscle. To unmask the direct effects of insulin on Nur77 and NOR1 protein expression without ß‐adrenergic stimulation, we utilized primary human skeletal muscle cells in culture. Skeletal muscle myoblasts (Lonza, Walkersville, MD) were isolated from a young healthy donor, seeded into standard 100 mm dishes at a density of 3500 cells/cm^2^ and maintained in skeletal muscle growth medium (SKGM; Lonza) supplemented with 0.1% human epidermal growth factor, 1% fetuin, 1% bovine serum albumin, 0.1% dexamethasone, 1% insulin and 0.1% gentamycin/amphotericin B. Cells were kept in a humid atmosphere at 37°C and 5% CO_2_ during all stages of the experimental process. When cells reached ~90% confluence, growth medium was replaced with differentiation medium (DMEM‐F12 supplemented with 2% horse serum and 1% antibiotic). Differentiation medium was refreshed every other day for 3 to 5 days until multinucleated myotubes were observed throughout the culture. Following differentiation, cells were returned to SKGM supplemented with insulin to 300 pM for 0, 30, and 180 min. We selected 300 pM insulin because it mimics postprandial hyperinsulinemia and is consistent with concentrations observed during the hyperinsulinemic‐euglycemic clamp procedure. However, Fu et al. (Fu et al. 2007) showed that insulin (100 nmol/L) treatment maximally induced NR4A3 mRNA at 2 h. After the completion of experimental conditions, cells were harvested by the cell scraping method into ice cold cell lysis buffer (#9803S, Cell Signaling) and prepared for immunoblotting as described above.

### Statistical analysis

Statistical analyses were performed using PRISM (GraphPad Software, La Jolla, CA). Data were tested for normality using the Shapiro–Wilk normality test. Datasets that were not normally distributed were log10 transformed for parametric analyses. A one‐way repeated measures ANOVA was used to test differences between groups (LHC vs. OB vs. T2DM). Paired t‐tests were used for comparison of gene and protein expression before and after the exercise training intervention. Pearson's correlation was used to investigate relationships among gene and protein targets and markers of metabolic health. Data are expressed as mean ± SEM. Statistical significance was accepted at *P* < 0.05.

## Results

### Baseline characteristics of the cross‐sectional cohort

Baseline characteristics of the cross‐sectional cohort are presented in Table 2. By design, the OB and T2DM subjects were older, weighed more, maintained a higher BMI and were more insulin resistant compared to the LHC participants. However, age and BMI were matched between OB and T2DM subjects.

**Table 2:** Subject characteristics of the cross‐sectional cohort

	LHC	OB	T2DM
*N* (sex)	14 (6 m, 8f)	12 (7 m, 5f)	10 (5 m, 5f)
Age (yrs.)	31 ± 1	66 ± 1[Link]	62 ± 3[Link]
Weight (kg)	64 ± 3	100 ± 4[Link]	94 ± 5[Link]
BMI (kg/m^2^)	22 ± 1	35 ± 1[Link]	33 ± 1[Link]
FPG (mg/dL)	91 ± 1	101 ± 2[Link]	125 ± 9[Link] ^,^ [Link]
GDR (“M,” mg/kg/min)	6.4 ± 0.5	2.5 ± 0.3[Link]	2.7 ± 0.4[Link]
Insulin Sensitivity (“M/I”) (mg/kg/min/*μ*U.mL)	0.115 ± 0.010	0.027 ± 0.004[Link]	0.025 ± 0.005[Link]

Data represent mean ± SEM.

LHC, lean healthy control subjects; OB, obese individuals with insulin resistance; T2DM, individuals with Type 2 Diabetes Mellitus; BMI, Body Mass Index; FPG, fasting plasma glucose; GDR, glucose disposal rate as calculated from the hyperinsulinemic‐euglycemic clamp; M/I, clamp‐derived glucose disposal rate normalized to prevailing insulin concentration; ^1^different from LHC; ^2^different from OB; one‐way ANOVA with Bonferroni *post hoc* test *P* < 0.05.

### Nur77 and NOR1 protein response to insulin in the cross‐sectional cohort

**Figure 1:**
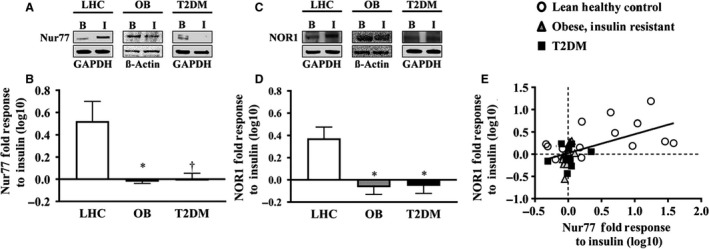
Skeletal muscle Nur77 and NOR1 protein expression. (A) Representative Western blot image for Nur77. (B) Nur77 is robustly induced by insulin stimulation in LHC, whereas this effect is blunted in OB and T2DM. (C) Representative Western blot image of NOR1. (D) NOR1 is robustly induced by insulin stimulation in LHC, whereas this effect is blunted in OB and T2DM. (E) Correlation between Nur77 and NOR1 protein response to insulin. LHC, lean healthy control subjects; OB, obese individuals with insulin resistance; T2DM, individuals with type 2 diabetes mellitus; B, Basal, *t* = 0 min; I, Hyperinsulinemia, *t* = 120 min. *, significant difference from LHC; one‐way ANOVA with Bonferroni *post hoc P* < 0.01; †, significant difference from LHC; one‐way ANOVA with Bonferroni *post hoc P* < 0.05. Data presented has been log10 transformed for normality.

**Figure 2:**
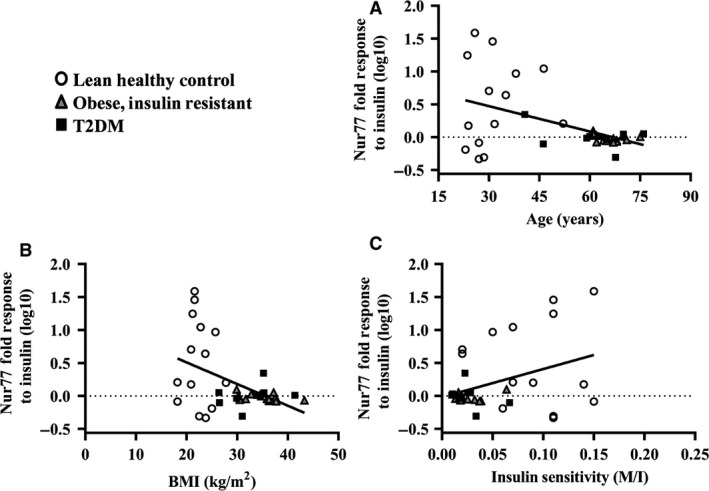
Baseline Nur77 correlations. The increase in Nur77 protein expression in response to insulin stimulation (“response to insulin”) negatively correlated with age (A) and BMI (B) and was positively associated with insulin sensitivity (“M/I” (mg/kg/min/*μ*U.mL), C). A log10 transformation was used to normalize the Nur77 data.

**Figure 3:**
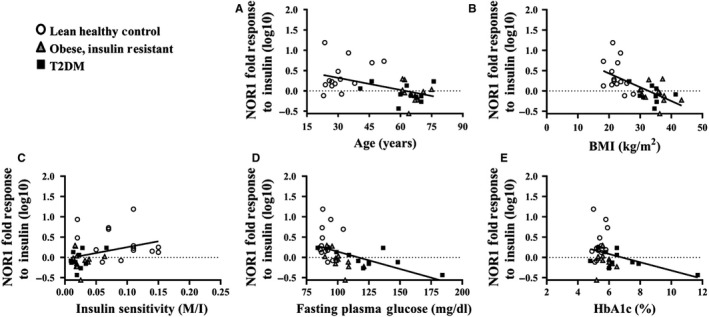
Baseline NOR1 correlations. The increase in NOR1 protein expression in response to insulin stimulation (“response to insulin”) was negatively correlated with age (A) and BMI (B) and was positively associated with insulin sensitivity (“M/I” (mg/kg/min/*μ*U.mL), (C) and improved glucose control (fasting plasma glucose, D; HbA1c, E). A log10 transformation was used to normalize the Nur77 data.

Skeletal muscle Nur77 and NOR1 protein expression increased in response to insulin stimulation in LHC (Nur77: 8.7 ± 3.2‐fold, *P* < 0.01; NOR1: 3.6 ± 1.1‐fold, *P* < 0.05), but remained unchanged in OB (Nur77: 1.0 ± 0.3‐fold, *P* > 0.05; NOR1: 1.0 ± 0.1‐fold, *P* > 0.05) and T2DM (Nur77: 1.1 ± 0.1‐fold, *P* > 0.05; NOR1: 1.0 ± 0.1‐fold, *P* > 0.05) **(**Fig. 1A–D**)**. Correlational analysis of all participants showed individuals had similar Nur77 and NOR1 inductions with insulin stimulation (*r* ^2^ = 0.34, *P* < 0.001, Fig. 1E). Correlational analysis was also used to investigate potential relationships between the Nur77 and NOR1 response to insulin and markers of obesity and metabolic health. The Nur77 response to insulin was inversely correlated with age (*r* ^2^ = 0.22, *P* < 0.01, Fig. 2A) and BMI (*r* ^2^ = 0.21, *P* < 0.01, Fig. 2B), but was positively correlated with clamp‐derived insulin sensitivity (M/I; *r* ^2^ = 0.14, *P* < 0.05, Fig. 2C). Similarly, the NOR1 response to insulin was inversely correlated with age (*r* ^2^ = 0.25, *P* < 0.01, Fig. 3A), BMI (*r* ^2^ = 0.42, *P* < 0.01, Fig. 3B), FPG (*r* ^2^ = 0.28 *P* < 0.01, Fig. 2D) and HbA_1c_ (*r* ^2^ = 0.12, *P* < 0.05, Fig. 2E), but was positively correlated with clamp‐derived insulin sensitivity (M/I; *r* ^2^ = 0.12, *P* < 0.05, Fig. 2C).

### Physiologic and metabolic effects of the lifestyle intervention

Data describing the effects of the exercise intervention are detailed in Table 3. As a result of the intervention subjects reduced bodyweight (Δ −8.5 ± 1.2 kg, *P* < 0.0001) and BMI (Δ 3.0 ± 0.4 kg/m^2^, *P* < 0.0001) while maintaining FFM (Δ −0.2 ± 0.8 kg, *P* > 0.05) and improving aerobic capacity (Δ 6.6 ± 0.9 mL/kg/min, *P* < 0.0001) and clamp‐derived GDR (Δ 1.6 ± 0.4 mg/kg/min, *P* < 0.0001). Concurrently, skeletal muscle glycogen content increased at basal (Δ 10.6 ± 8.8 mmol/kg wet wt.) and insulin‐stimulated conditions (Δ 44.4 ± 6.8 mmol/kg wet wt.).

**Table 3:** Metabolic characteristics of the lifestyle intervention cohort

	PRE	POST
*N*	18 (11 m, 7f)	18
Age (yrs.)	66 ± 1	–
Weight (kg)	97.9 ± 3.7	89.5 ± 3.1[Link]
BMI (kg/m^2^)	34.5 ± 1.0	31.5 ± 0.9[Link]
Fat Free Mass (kg)	56.3 ± 2.7	56.1 ± 2.7
*V*O_2max_ (mL/kg/min)	21.6 ± 0.8	28.1 ± 1.5[Link]
FPG (mg/dL)	109 ± 4	101 ± 3[Link]
GDR (mg/kg/min)	2.6 ± 0.3	4.2 ± 0.3[Link]
M/I (mg/kg/min/*μ*U.mL)	0.024 ± 0.003	0.041 ± 0.005[Link]
RQ (AU)	0.84 ± 0.01	0.83 ± 0.01
Metabolic Flexibility (AU)	0.03 ± 0.01	0.06 ± 0.01[Link]
Fat Oxidation_Basal_ (mg/kg FFM/min)	1.11 ± 0.10	1.10 ± 0.12
Fat Oxidation_Insulin_ (mg/kg FFM/min)	0.76 ± 0.08	0.57 ± 0.07[Link]
COX (mg/kg FFM/min)	2.06 ± 0.24	1.95 ± 0.26
NOGD (mg/kg FFM/min)	1.7 ± 0.6	4.1 ± 0.6[Link]
Glycogen Content_Basal_ (mmol/kg wet wt.)	88 ± 10	105 ± 5[Link]
Glycogen Content_Insulin_ (mmol/kg wet wt.)	102 ± 9	155 ± 8[Link]

Data represent mean ± SEM.

The OB and T2DM subjects from the cross‐sectional analysis have been combined into PRE and POST, which represents data collection points before and after the intensive lifestyle intervention; BMI, Body Mass Index; GDR, Glucose Disposal Rate as calculated from the hyperinsulinemic‐euglycemic clamp; *V*O_2_max, maximal oxygen consumption during maximal aerobic exercise testing; COX, Carbohydrate Oxidation; NOGD, Nonoxidative Glucose Disposal; BMR, Basal Metabolic Rate; RQ, Respiratory Quotient; FPG, Fasting Plasma Glucose; M/I, GDR normalized to the prevailing insulin concentration during the final 90–120 min of the hyperinsulinemic‐euglycemic clamp (mg/kg/min/μU.mL); ^1^significant effect of the 12‐week intensive lifestyle intervention; paired sample t‐test, *P* < 0.05.

### Nur77 and NOR1 gene and protein expression with chronic aerobic exercise training

**Figure 4:**
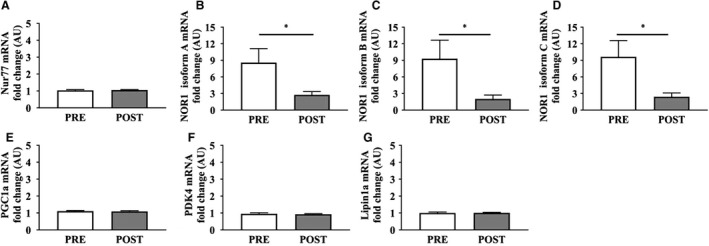
Skeletal muscle gene expression before and after exercise training. (A) Nur77 gene expression in response to insulin stimulation was unchanged after exercise training. (B–D) The NOR1 Isoform A, B, and C response to insulin was significantly reduced after exercise training. (E–G) PGC1*α*, PKD4, and lipin1*α* gene expression in response to insulin stimulation was unchanged after exercise training. *, *P* < 0.05.

Skeletal muscle gene expression for Nur77 and three isoforms of NOR1 (Isoform A, B, C) were analyzed at basal and under insulin‐stimulated conditions, before and after the aerobic exercise training intervention **(**Fig. 4A–D). Nur77 gene expression remained unchanged in response to insulin following the exercise intervention (Fig. 4A). All three NOR1 isoforms showed an attenuated response to insulin following exercise training **(**Fig. 4B–D). Known skeletal muscle regulatory targets (Wang and Muscat 2013) (PPAR‐y coactivator‐1*α*, PGC1*α*; pyruvate dehydrogenase kinase‐4, PDK4; lipin1*α*; Fig. 4E–G) were also analyzed at basal and under insulin‐stimulated conditions, before and after the exercise intervention. The intervention did not have any significant effect on skeletal muscle PGC1*α*, PKD4, or lipin1*α* gene expression in response to insulin.

**Figure 5:**
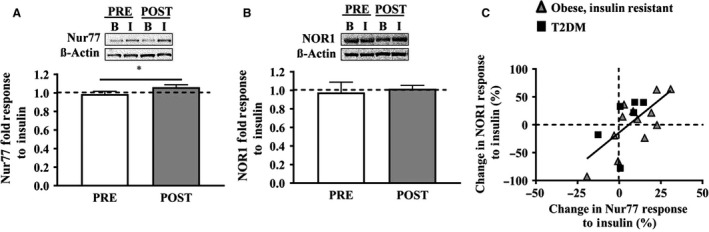
Nur77 and NOR1 protein response to insulin before and after exercise training. (A) The Nur77 protein expression in response to insulin modestly improved from PRE to POST exercise training (PRE: −1.2 ± 0.3%, POST: +6.2 ± 1.5%, *P* = 0.024), whereas the NOR1 response to insulin remained unchanged (B). With aerobic exercise training, changes in the Nur77 response to insulin were associated with changes in the NOR1 response to insulin (C).

To determine whether the Nur77 and NOR1 gene responses translated into functional proteins, we analyzed skeletal muscle protein expression before and after the lifestyle intervention. Chronic aerobic exercise training modestly increased Nur77 protein expression in response to insulin stimulation (Fig. 5A; PRE: −1.2 ± 0.3%, POST: 6.2 ± 1.5%, *P* = 0.024), whereas NOR1 showed a similar trend, but remained statistically unaffected (Fig. 5B). The changes observed in Nur77 protein response to insulin after exercise training were associated with changes in NOR1 protein response to insulin after exercise training (Fig. 5C), in agreement with our cross‐sectional characterization.

### Cell culture validation study

**Figure 6:**
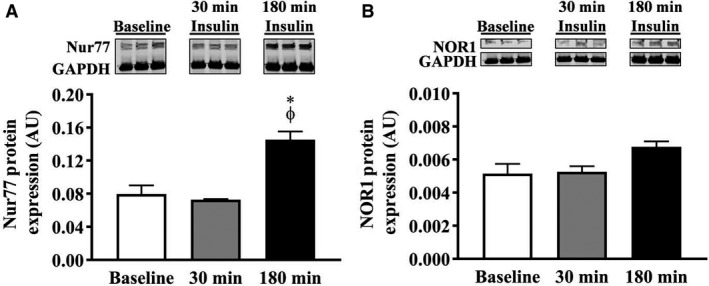
Cell culture validation study. Human skeletal muscle cells were treated with 300 pM insulin for 0, 30, and 180 min. (A) Nur77 protein expression was unchanged at 30 minutes, but significantly increased at 180 min (one‐way ANOVA,* P* = 0.0016). (B) NOR1 protein expression showed a similar trend, but did not reach significance (1‐way ANOVA,* P* = 0.0701). *, Bonferroni *post hoc P* < 0.05 compared to baseline, Φ, *P* < 0.05 compared to 30 min.

We conducted a cell culture validation study to verify the normal physiologic response to insulin on Nur77 and NOR1 protein expression in human skeletal muscle myotubes. In response to 300 pM insulin, Nur77 protein expression remained unchanged at 30 minutes, but was significantly increased at 180 min (Fig. 6A; 183 ± 12.6% compared to baseline, 1‐way ANOVA, *P* = 0.002; Bonferroni's Multiple Comparison *post hoc* test, *P* < 0.05.) NOR1 protein expression displayed a similar trend, but with lesser magnitude did not reach statistical significance at 180 minutes **(**Fig. 6B, **131 **±** **6.3% compared to baseline, 1‐way ANOVA, *P* = 0.070). These results suggest insulin stimulates Nur77 and NOR1 protein expression in human skeletal muscle cells independent of the ß‐adrenergic effect of insulin.

## Discussion

To our knowledge, these are the first data to show that skeletal muscle Nur77 and NOR1 protein expression is blunted in response to insulin in obesity and overt T2DM, and that aerobic exercise training improves this response. Since insulin is an important physiological regulator of glucose and fatty acid metabolism, we focused our investigation on the changes in response to insulin stimulation during the hyperinsulinemic‐euglycemic clamp. Glucose and lipid metabolism are known to be dysregulated in obesity, insulin resistance, and T2DM. Therefore, understanding the major control points underlining these metabolic derangements may lead to the development of novel therapies for the prevention and treatment of obesity and T2DM. Previous research from cell culture and animal models suggest an important role for both Nur77 and NOR1 in glucose and lipid metabolism (4‐8). While preclinical and human studies have described defects in Nur77 and NOR1 in obesity (4; 9‐11), the data herein deepen our understanding of how skeletal muscle Nur77 and NOR1 may function across a range of human phenotypes: LHC, to OB and T2DM. Furthermore, the associations between the Nur77 and NOR1 protein response to insulin, with multiple measures of metabolic function, provides additional incentive to investigate Nur77 and NOR1 in the context of insulin resistance and T2DM.

An important finding from the cross‐sectional analysis was the remarkable blunting of the Nur77 and NOR1 response to insulin, which occurred to a similar extent in both the OB and T2DM groups compared to LHC. This suggests dysregulated Nur77 and NOR1 signaling may present early in the natural history of diabetes and likely reaches a conceptual “floor” in early insulin resistance. Given this early‐onset blunting, Nur77 and NOR1 present an intriguing therapeutic target to prevent the progression from obesity (a prediabetic, insulin resistance state) to overt T2DM. Furthermore, we conducted a subgroup analysis of the OB participants, a group that exhibits the most heterogeneity related to metabolic health across the spectrum, spanning healthy physiology to overt T2DM. This analysis revealed that poorer glucose control (fasting plasma glucose) was associated with reduced Nur77 (*r* ^2^ = 0.45, *P* = 0.017, *n* = 12) and NOR1 (*r* ^2^ = 0.40, *P* = 0.028, *n* = 12) response to insulin. In agreement with data from the full cohort, the relationship between insulin sensitivity (M/I) and the Nur77 and NOR1 response to insulin, data from the OB group suggest that metabolic health and the Nur77 and NOR1 protein response to insulin are altered in unison across the glucose tolerance continuum and follow the natural history of diabetes. Future research should investigate if normalizing the Nur77 and NOR1 response to insulin in obese, insulin‐resistant individuals (prediabetic) can maintain or improve insulin sensitivity and prevent the progression toward overt T2DM.

While we did not examine the specific mechanisms behind these observations, current preclinical literature in this area may provide insight into why obesity and insulin resistance attenuate orphan nuclear receptor responses to insulin. For example, Nur77 and NOR1 are transcriptional regulators of glucose metabolism and downstream effectors of β‐adrenergic receptor signaling (Coletta et al. 2008). Thus, we speculate that the early‐onset of insulin resistance and impaired metabolic flexibility is directly linked to Nur77 and NOR1 down regulation, which subsequently exacerbates impaired metabolic flexibility.

In addition to the cross‐sectional comparison between LHC, OB, and T2DM, we performed a therapeutic lifestyle intervention in OB and T2DM participants involving aerobic exercise training. Our selection of this intervention was twofold: aerobic exercise training is a well‐documented countermeasure to obesity and T2DM that confers benefit through skeletal muscle adaptations, and aerobic exercise training has been previously shown to stimulate Nur77 and NOR1 gene expression (Chao et al. 2009; Mey et al. 2017). We observed a significant effect of exercise training on skeletal muscle gene expression of NOR1 isoforms A, B, and C, whereas Nur77 gene expression was unchanged after exercise training. These gene expression changes were not mirrored in our analysis of Nur77 and NOR1 protein expression, as Nur77 protein expression increased with insulin stimulation only after the aerobic exercise training intervention. Our basal gene expression data corroborate previous findings from microarray analysis in combination with the hyperinsulinemic‐euglycemic clamp (Rome et al. 2003; Wu et al. 2007; Coletta et al. 2008; Kanzleiter et al. 2010). In contrast to our hypothesis, we observed a marked attenuation in insulin‐stimulated NOR1 isoform A, B, and C gene expression. We generated our hypothesis based on cell culture, murine, and human microarray data evidencing the stimulatory effect of exercise on Nur77 and NOR1 gene expression. However, the prior human studies were performed in untrained, healthy adults, and skeletal muscle samples were obtained within 3 h of exercise (Mahoney and Schwartz 2005; Mahoney et al. 2005). It is possible that the insulin‐resistant skeletal muscle in OB and T2DM subjects present with translational repression of Nur77 and NOR1 (Rodriguez‐Calvo et al. 2017). To compensate for this inability to translate the gene responses into functional protein, OB and T2DM skeletal muscle may bolster Nur77 and NOR1 gene expressions in response to insulin stimulation, without concomitant increases in protein expression. The reductions observed in NOR1 gene expression in response to insulin stimulation may then represent a movement toward a healthier phenotype, and is in line with our Nur77 and NOR1 protein observations. Still, there remains a clear deficiency in Nur77 and NOR1 gene and protein regulation in response to insulin in OB and T2DM, even after the advent of a chronic aerobic exercise intervention. This aligns with other research showing that individuals with T2DM have epigenetic modifications that reduce Nur77 gene expression and inhibit insulin signaling (Chen et al. 2016). Still, this requires additional investigation and will improve the understanding of Nur77 and NOR1 physiology. Prior research utilizing acute aerobic exercise has shown increases in basal Nur77 and NOR1 (Goode et al. 2016) protein expression. However, we did not observe differences in basal expression of Nur77, NOR1 or the putative transcriptional targets (PDK4, PGC1α, LIPIN1α) after the exercise intervention, which is consistent with others (Mahoney et al. 2005). This may have arisen because our sample acquisition occurred > 48 h after the final bout of exercise training, a timeline that represents a critical control point for our metabolic measures, but prevents the observation of an acute effect of aerobic exercise on skeletal muscle Nur77 and NOR1. Whether the acute stimulation of Nur77 and NOR1 skeletal muscle gene expression observed by others in lean, healthy individuals is blunted in obesity or T2DM remains to be elucidated. Nevertheless, we observed modest increases in the Nur77 protein response to insulin after aerobic exercise training, which indicates an improvement toward a lean, healthy phenotype and mirrors the metabolic improvements in response to insulin imparted by the lifestyle intervention used in this study. This trend toward a lean, healthy phenotype without complete normalization is an understandable result, as the exercise participants were still older, overweight, and insulin resistant compared to LHC. Whether the Nur77 or NOR1 gene and protein response to insulin can be fully normalized with continued exercise training remains to be determined, but represents a promising area for future research, for example, pharmacologic targeting of orphan nuclear receptors.

Finally, we performed a validation study to examine the independent effect of insulin stimulation in human skeletal muscle cells. Prior research in rat and mouse skeletal muscle has focused on Nur77 and NOR1 gene expression, primarily in response to ß‐adrenergic stimulation utilizing isoprenaline (Pearen et al. 2006, 2008; Myers et al. 2009). Furthermore, it has been documented that in vivo insulin stimulation imparted by the hyperinsulinemic‐euglycemic clamp procedure induces a ß‐adrenergic response (Young et al. 2010). Therefore, we were interested in investigating the Nur77 and NOR1 protein expression in response to insulin stimulation utilizing a human skeletal muscle cell culture model, which removes the confounding ß‐adrenergic response to insulin stimulation observed in vivo. Our results indicate an independent effect of insulin stimulation on Nur77 and NOR1 protein expression after 180 min of physiologic insulin stimulation. Taken together with our in vivo human data, the results are promising and suggest that Nur77 and NOR1 may be partly responsible for the physiologic differences in response to insulin stimulation observed in OB and T2DM individuals.

### Limitations

We utilized a lean, healthy control group as a measure of optimal health to understand normal Nur77 and NOR1 physiology in response to insulin. By design, the control group was younger, more fit and more insulin sensitive than the OB and T2DM groups. Although differences in age limits our ability to distinguish the primary driver of the blunted Nur77 and NOR1 response to insulin in OB and T2DM, the data do provide clear evidence of a deviation from healthy physiology in our obese and diabetic cohorts. Furthermore, we confirmed that the relationship between both the Nur77 or NOR1 protein response to insulin and insulin sensitivity (M/I), persists after controlling for age (Nur77: *r* ^2^ = 0.22, adjusted *r* ^2^ = 0.18, *P* = 0.015; NOR1: *r* ^2^ = 0.65, adjusted *r* ^2^ = 0.63, *P* < 0.0001). Still, future research on the Nur77 and NOR1 response to insulin stimulation should utilize an age‐matched control group to better assess the impact of obesity or T2DM *per se*. These results are exciting given that the lifestyle intervention in OB and T2DM increased the Nur77 response to insulin, suggesting a return toward a healthy physiological state. The lifestyle intervention involved aerobic exercise training and nutritional caloric restriction which elicited weight loss and improved insulin sensitivity. Although we are unable to fully differentiate the independent effects of these primary lifestyle factors (aerobic fitness and weight loss), correlational analysis suggests neither aerobic fitness nor weight loss is related to the Nur77 or NOR1 protein response to insulin. Collectively, these data show that a lifestyle intervention in OB and T2DM initiates a return toward healthy skeletal muscle physiology, which is mirrored by changes in skeletal muscle Nur77 and NOR1. On a technical note, we were forced to use two different primary antibodies and loading controls in this study due to unforeseen problems with our antibody supplier and detection method. Thus, we displayed our protein expression data as fold change to examine insulin responsiveness, which was the focus of this investigation in any event.

## Conclusion

The data provide evidence that Nur77 and NOR1 insulin responsiveness is blunted in obesity and T2DM, and that a lifestyle intervention involving aerobic exercise training partially recovers this response. This research provides an extension of current knowledge (Pearen and Muscat 2018), and provides incentive to investigate the therapeutic potential of Nur77 and NOR1 for the treatment and prevention of T2DM. Further mechanistic work to understand the precise regulation of these signaling molecules will help to achieve this therapeutic potential.

## Conflict of Interest

There are no conflicts of interest.
